# Epigenetics and Vascular Senescence–Potential New Therapeutic Targets?

**DOI:** 10.3389/fphar.2020.535395

**Published:** 2020-09-29

**Authors:** Qian Ding, Chunhong Shao, Peter Rose, Yi Zhun Zhu

**Affiliations:** ^1^State Key Laboratory of Quality Research in Chinese Medicine and School of Pharmacy, Macau University of Science and Technology, Macau, China; ^2^School of Basic Medicine, Guizhou University of Traditional Chinese Medicine, Guiyang, China; ^3^Department of Psychiatry, Huashan Hospital, Fudan University, Shanghai, China; ^4^School of Biosciences, University of Nottingham, Loughborough, United Kingdom

**Keywords:** vascular aging, epigenetics, cell senescence, inflammation, oxidation stress, calcification

## Abstract

Epigenetics is defined as the heritable alterations of gene expression without changes to the coding sequence of DNA. These alterations are mediated by processes including DNA methylation, histone modifications, and non-coding RNAs mechanisms. Vascular aging consists of both structural and functional changes in the vasculature including pathological processes that drive progression such as vascular cell senescence, inflammation, oxidation stress, and calcification. As humans age, these pathological conditions gradually accumulate, driven by epigenetic alterations, and are linked to various aging-related diseases. The development of drugs targeting a spectrum of epigenetic processes therefore offers novel treatment strategies for the targeting of age-related diseases. In our previous studies, we identified HDAC4, JMJD3, Fra-1, and GATA4 as potential pharmacological targets for regulating vascular inflammation, injury, and senescence.

## Introduction

In the 19th century, the father of modern medicine William Osler stated, “a man is only as old as his arteries.” During vascular aging, pathological processes drive changes in the structure and function of blood vessels including dysregulation in vascular homeostasis and vascular remodeling, leading to lumen dilation, vascular stiffness, and thickening. At the molecular level dysregulation in vascular homeostasis is promoted by vascular cell senescence, widespread inflammation, oxidation stress, and calcification (Ding et al., 2018). It is now widely recognized that vascular aging is intimately linked with cardiovascular diseases (CVD) including atherosclerosis (AS), hypertension, coronary heart disease, and stroke (Lakatta and Levy, 2003b). Aside from high mortality rates, CVD also leads to reduce quality of life in afflicted individuals and high burden on society and families (Van Camp, 2014).

Epigenetics is defined as processes that governs the expression of a gene(s) without altering the sequence of coding DNA. These heritable changes in expression are controlled by distinct chemical modifications to bases present in DNA including DNA methylation and histone modification in addition to non-coding RNA (ncRNA) mechanisms. In healthy tissues, normal gene expression occurs as a result of interactions between genetic and environmental factors *viz*. smoking, obesity, or alcohol consumption, which can cause dysregulation in cellular homeostasis, having negative impacts on health. Epigenetics changes can explained many of the interaction between genes and environment cues and can explain altered risk of developing diseases in humans (Feinberg, 2018). Indeed, as age increases the cumulative effects of stress and environmental impacts promotes the gradual accumulation of epigenetic changes in tissues. These epigenetic changes could serve by increasing an individuals susceptibility and risk of developing chronic diseases. Fortunately, many of these epigenetic modifications can be reversed, and targeting the respective enzymes that control methylation or histone modifications has been proposed as useful drug target in the treatment of age-related diseases. Therefore, in the current review, coverage of vascular aging and associated epigenetics processes will be covered. In addition, a summary of the main pathological drivers of vascular cell senescence, inflammation, oxidation stress, and calcification will be provided. A better perspective of epigenetic changes that occur during vascular aging will help to better understand the process of vascular damage that occurs with age. This information could be used to better development therapeutics or strategies to delay or treat aging-related cardiovascular diseases (Sen et al., 2016).

## Vascular Aging

**Figure 1:**
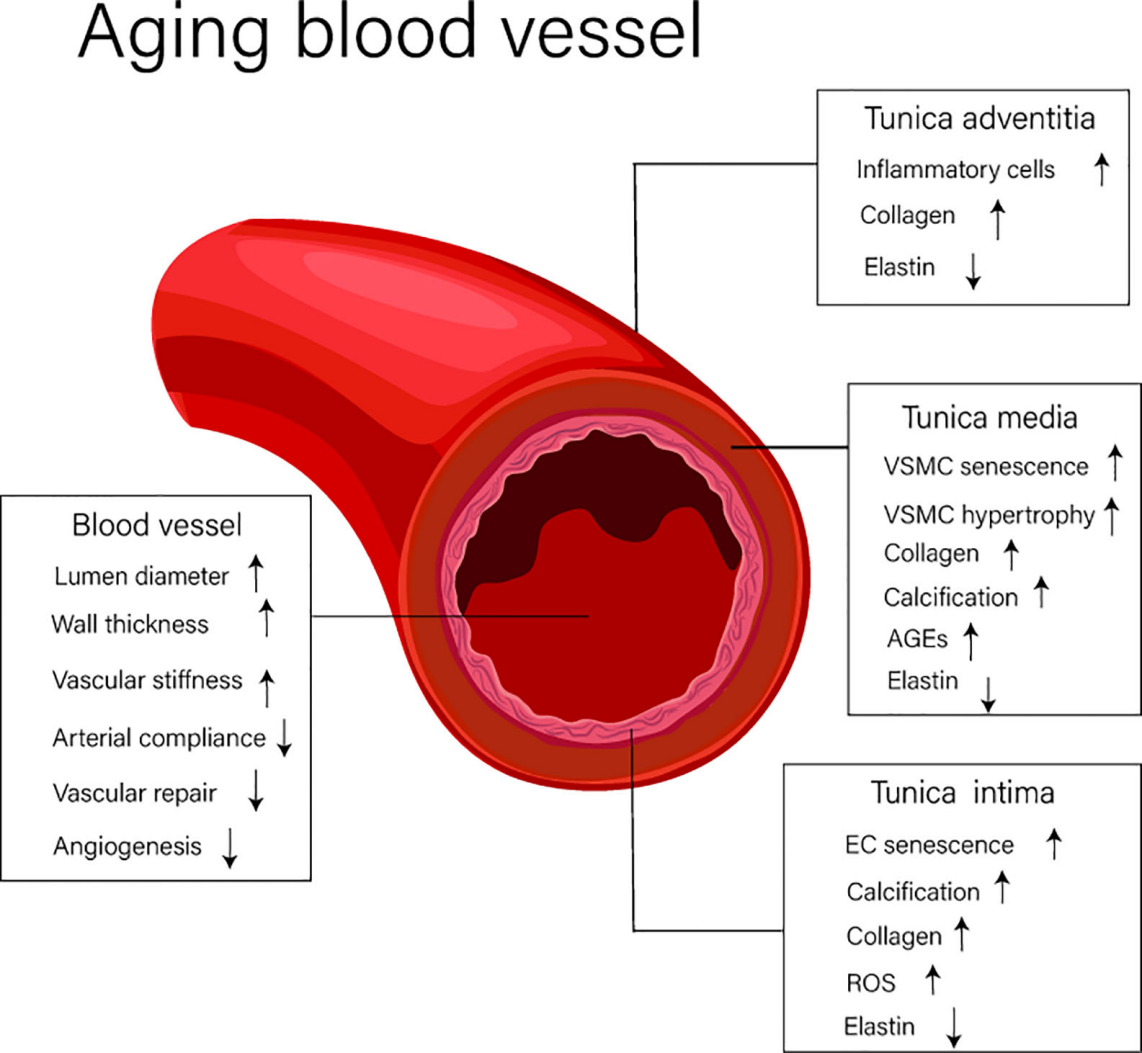
The structure and functional changes of blood vessels. EC, endothelial cell; VSMC, vascular smooth muscle cell; AGEs, advanced glycation end products; ROS, reactive oxygen species.

Vascular aging is characterized by changes in both structural and functional elements associated with blood vessels. Over the course of time, vascular aging leads to lumen dilation, vascular stiffness, and thickening, these changes being largely driven by pathological processes including vascular cell senescence, widespread inflammation, oxidation stress, and tissue calcification (Ding et al., 2018). The structural and functional changes of blood vessels that occurs during vascular aging are shown in **Figure 1**.

### Structural Changes

Blood vessels (excluding capillaries) are composed of distinct anatomical features comprising the intima, media, and adventitia. The intima is largely composed of endothelial cells (ECs) and is the first defensive layer important in mitigating the development of vascular diseases. The media consists of vascular smooth muscle cells (VSMCs), elastic fibers, and extracellular matrix, and the adventitia, the outermost layer, is composed of loose connective tissue. This region is consists of thick collagen fibers and disordered elastin fibers. It is widely known that during aging, significant change occur in the intima, and this alters the function properties of this layer and, importantly, how the intima interacts with adjacent regions like the media (Lakatta et al., 2009). These changes promote altered responses to luminal dilation, vascular stiffness, and the thickening of blood vessels. Indeed, research has shown that the diameter of the aorta of elderly people, those over 65 years of age, increases by 15–20% compared with that of tissues from younger individuals (Lakatta, 2003). In addition, increased thickening of the arterial wall, largely driven by thickening of the arterial intima and media (Lakatta and Levy, 2003a), is associated with increased abundance of hypertrophic smooth muscle cells. Other common structural changes associated with aged blood vessels include elastin breaks, increased collagen abundance, and elevated levels of advanced glycation end products (AGEs). Combined these changes have a dramatic impact on the severity of vascular aging (Lakatta, 2003; Lakatta and Levy, 2003a).

### Functional Changes

Increased arterial stiffness, decreased arterial compliance (AC), reductions in vascular repair, and diminished capacity to control processes like angiogenesis are important features of aging blood vessels. Arterial stiffness and decreased AC can be attributed to smooth muscle cell hypertrophy, arterial calcification, and ECM remolding. One of the key reasons for the decline in the ability of vessels to repair tissue damage is thought to be due to vascular endothelial cell senescence (Lakatta and Levy, 2003a; Novella et al., 2012).

### Pathological Process During Vascular Aging and Cardiovascular Diseases

Aging blood vessels promote the development of vascular diseases and in turn accelerates the process of vascular aging. Numerous epidemiological studies indicate that lipid levels, diabetes, sedentary lifestyles, and various genetic factors increase the risk of coronary heart disease, hypertension, heart failure, and stroke. Underpining these changes are distinct biochemical and physiological changes that drive changes in the cardiovascular system. In recent times, the conventional cardiovascular continuum (CCC) in 2006 (Dzau et al., 2006a; Dzau et al., 2006b) has proposed that CVD begins with risk factors and progresses to terminal stage cardiac disease through a series of steps. The main characteristics of CCC are viewed as coronary artery atherosclerosis (AS), leading to coronary stenosis, and myocardial ischemia and myocardial infarction. However, one aspect not considered by the CCC criteria is the role of aging, especially vascular aging in the occurrence and development of CVD. Therefore, in 2010, the aging cardiovascular continuum (ACC) suggested that a key component of CVD should include aging (O’Rourke et al., 2010). Therefore, the ACC describes the stages of cardiovascular disease (CVD) as vascular aging, which promotes aorta dilation and sclerosis. In turn, these pathoglogical changes lead to heart failure, terminal stage cardiac disease, and death. In essence, the basic characteristics proposed by the ACC are the progressive degradation of the proximal aorta with arterial dilatation and sclerosis that has an adverse effects on the heart. If we consider that aortic pulsation, then the repetitive stretching and relaxation of arteries has occured 3 billion times in elderly people over 80 years old; it is clear that aging is an important factor in this process. As such, aging should be viewed as a risk factor for CVD, since it explains 50% of all clinical CVD cases in senior citizen (Cunha et al., 2017).

Vascular aging and CVD have several common pathological features when viewed at the molecular and cellular levels *viz*. increased oxidative stress, a pro-inflammatory environment, dysregulation in cell signaling, and altered response to infiltrating immune cell types. These feactures, as mentioned, provide sufficient conditions in the aged artery for the development of cardiovascular disease. For example, arterial aging and atherosclerosis have similar structural and biochemical characteristics. Indeed, research has shown that abnormal plasma cholesterol levels of young people is related to arterial wall thickening, VSMCs, collagen proliferation, and collagen deposition. These changes are similar to those observed in elderly people with altered cholesterol levels (McGill et al., 2008; Wang et al., 2010a). However, some animal studies show that plasma lipid levels do not change with aging; however, the prevalence, severity, and negative impact of atherosclerosis still increase in aged animals (Eto et al., 2008). These observations suggesting other molecular events are also important in driving CVD. One clue then may be that many proteins that are highly expressed in atherosclerotic tissues are also increased in aging arterial walls such as MFG-E8 and MMPs (Fu et al., 2009).

**Figure 2:**
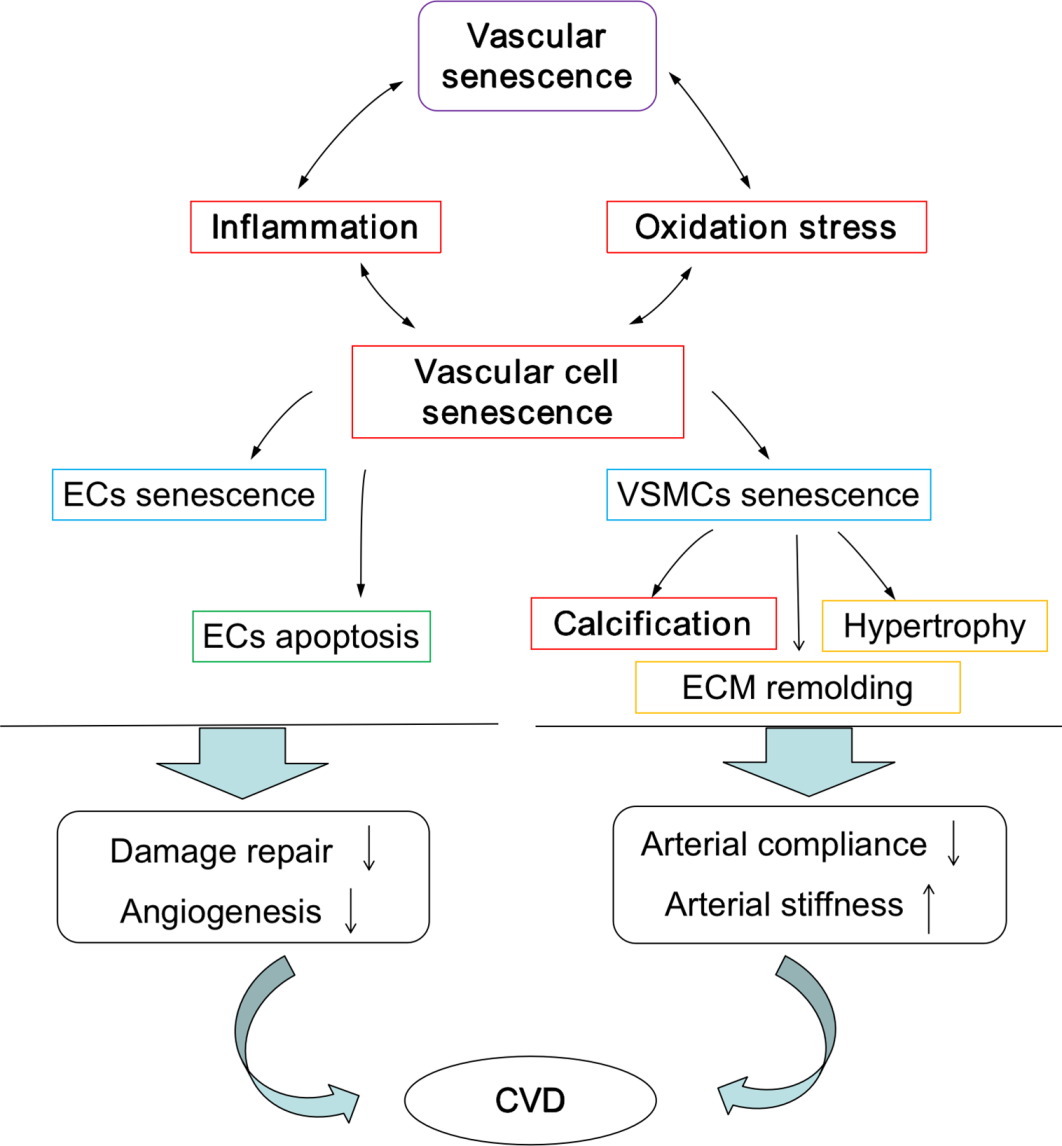
Pathological process during vascular aging. Pathological process promote the development of vascular aging and vascular aging accelerates the pathological process. EC, endothelial cell; VSMC, vascular smooth muscle cell; ECM, extracellular matrix; CVD, cardiovascular diseases.

The pathological processes assocociated with vascular aging are characterized by vascular cell senescence, widespread inflammation, oxidation stress, and calcification, and these processes are summarized in **Figure 2**. As ECs age, the cells become flattened and enlarged, the rates of proliferation is diminished, and rates of apoptosis increased. In addition, enrichment of the surrounding environment with inflammatory mediators including interleukins-6 and tumour necrosis factor-α results in the decreased ability to control vascular repair and angiogenesis. Similalrly, the numbers of hypertrophic VSMCs begin to increase, as do the rates of cell proliferation and migration, and combined, this cellular transition drives extracellular matrix (ECM) remolding. Critically, as rates of inflammation increases over time, so do changes in cell populations of EC and VSMCs, and this, in turn, stimulates vascular aging and ultimately the endothelium damage. A widely recognized key driver of chronic inflammation in the vasculariture, especially during aging, is the renin-angiotensin II (Ang II) signaling pathway. Changes in this signaling system initiates a cascde of events leading to the activation of downstream pro-inflammatory transcription factors like nuclear factor kappa beta (NF-kB), the production of reactive oxygen species (ROS) leading to oxidative damage, and the induction of endoplasmic reticulum (ER) stress. ER and sarcoplasmic reticulum are important pools of calcium; therefore, this could promote dysregulation in Ca^2+^ signaling. Collectively, these molecular events accelerate vascular remodeling (Cavallaro et al., 2000; Lakatta et al., 2009; Wang et al., 2010a; Wang et al., 2010b; Krebs et al., 2015). Under normal physiological conditions, ROS are produced in the vascular system through two routes, the mitochondrial and non-mitochondrial pathways. Importantly, in healthy or younger cells, oxidative stress is controlled by cells having the capacity to remove excess ROS *via* antioxidant system, and this could potentially reduce the rates of oxidative damage to mtDNA, respiratory chain complex proteins, and other important cellular components, therefore maintaining adequate cellular and tissue redox homeostasis (Kadlec et al., 2016). However, during aging, mitochondrial function can diminish, and this results in a cumulative increase of mitochondrial damage and excessive ROS production. Consequently, increased levels of ROS are generated *via* active oxygen release mechanism, resulting in further damage to the mitochondrial outer membrane and causing membranes to rupture, intracellular calcium overload, DNA damage, and the release of pro-apoptotic proteins like cytochrome C. This cascade ultimately leads to the induction of apoptosis or necrosis and further amplifies damage to aged or aging tissues fueling age-related degenerative diseases (Mikhed et al., 2015).

Tissue calcification is also common in the aged cardiovascular system, and researchers have demonstrated that senescent VSMCs are associated with a calcification phenotype. Aging VSMCs produce appreciable levels of calpain-1, and these promote blood vessels calcification, a process commonly referred to as biomineralization (Jiang et al., 2012). Calcification activates tissue transglutaminase (TG2) and upregulates calcification promoter genes such as the osteoblast transcription factor Runt-related transcription factor 2 (Runx2) and bone morphogenetic protein-2 (BMP-2). These systems participate in driving arterial calcification and sclerosis in the aging blood vessel wall. It is now realized that vascular calcification is an important cause of the increase in the incidence and mortality of cardiovascular diseases (Leopold, 2013) and that calcification, VSMCs hypertrophy, ECM remolding promote arterial stiffness and decreased AC.

## Epigenetic Control of Gene Expression

Epigenetics is the heritable alterations of gene expression without changing the DNA sequence and determines whether a gene is turned on or off (Handy et al., 2011). Mounting evidence shows that epigenetic mechanisms play an important role in phenotypes and behavioral changes. Indeed, these mechanisms go someway to explain why twins who grew up in different locations and under differing environmental conditions, yet having the same genetic background, can have differences in lifespans or altered risks of chronic diseases such as diabetes or hypertension (Fraga et al., 2005; Tan et al., 2013). Similarly, dietary influences like caloric restriction can delay the occurrence of age-dependent diseases *via* epigenetic mechanisms. Again, these examples exemplify the intimate association between how environmental factors can drive changes in epigenetic events that control gene expression (Maegawa et al., 2017).

### DNA Methylation

DNA methylation, the direct addition of a methyl group to the 5^th^ carbon atom of cytosine, is one the most widely recognised epigenetic mechanims known to regulate gene expression. CpG islands, characterized as short interspersed DNA sequences that are GC-rich regions. In mammalian cells, most of these CpG regions can be methylated (Jeltsch, 2008). Importantly, *de novo* DNA methylation status is governed by a family of enzymes known as DNA methyltransferases that include DNMT3a and DNMT3b, along with DNMT1 that is important during replication (Okano et al., 1999). The activities of these enzymes are readily influenced by environmental cues making DNA methylation a dynamic process in cells and tissues (Tsaprouni et al., 2014). Generally speaking, high methylation rates in the gene promoter region of genes inhibit gene expression. Inhibition in this instance is caused by altered (reduced) transcription factors binding to promoters consensus regions or by reducing the recruitment of chromatin modifying enzymes (Kohli and Zhang, 2013). To date, a wide range of abnormal DNA methylation patterns have been characterized in aged cells, with many seen in various age-related diseases (Hai and Zuo, 2016).

### Histone Modifications

Histone modifications is another important epigenetic mechanism that regulates gene transcription by changing how histones proteins interact with DNA. Changes in DNA histone interactions is important in controlling gene expression during processes such as replication, transcription, and repair. Common mechanisms of histone modification include methylation, acetylation, phosphorylation, and ubiquitination. Unlike DNA methylation, the effect of histone modifications on gene expression may vary due to the specific type of chemical modifications (Shahbazian and Grunstein, 2007). Enzymes that regulate histone modifications include histone deacetylase (HDAC), histone methyltransferase, and histone acetyltransferase, and it is widely known that these enzymes play an important roles in the process of vascular aging (Calvanese et al., 2009).

### ncRNA Mechanisms

ncRNA is RNA that lacks the capacity to code for a protein. Examples of ncRNA include microRNA (miRNA), long ncRNA (lncRNA), and small interfering RNA (siRNA). Although ncRNA has no direct affect on chromatin structure, it does play an important role in post-transcriptional control of gene expression (Gurha and Marian, 2013). Genome-wide RNA sequencing shows that ncRNAs are differentially expressed in both senescent and normal cells (Wu et al., 2015; Anderson et al., 2016).

## Do Epigenetics Mechanisms Contribute to Vascular Aging?

The following introduces the pathological steps that are involved in the epigenetic regulation of vascular aging. A summary of these events and epigenetic targets are shown in **Table 1**.

**Table 1:** Vascular aging related epigentics targets.

Targets	Major findings	Effects	References
SIRT1	Decreases in VSMC of aged mice	Enhance vascular inflammation	(Chen et al., 2016)
	Deacetylate histone H4K16	Improves the function of endothelial cells	(Wan et al., 2014)
	Increase ECs KLF2 expressions	Vaso-protective	(Gracia-Sancho et al., 2010)
	Increased by energy limitation	Fight abdominal aortic aneurysm	(Liu et al., 2016)
	Activation by SIRT1 activators	Inhibit vascular remodeling, stiffness and calcification	(Takemura et al., 2011; Winnik et al., 2015; Fry et al., 2016; Badi et al., 2018)
SIRT2	Deacetylate p65Lys310	Regulates inflammmation *via* NF-κB-dependent gene expression	(Rothgiesser et al., 2019)
SIRT3	Missing will lead to the high acetylation and inactivation of SOD2	Leading to an imbalance of redox homeostasis in blood vessels	(Dikalova et al., 2017)
SIRT6	Protect telomere	Avoiding premature cell senescence caused by DNA damage	(Cardus et al., 2013)
HDAC3	Inhibit the activation of macrophages	Lacking HDAC3 will be easily activated by IL-4 and accelerate blood vessel’s inflammation	(Mullican et al., 2011)
HDAC4	Deacetylate FoxO3a	Regulates vascular inflammation *via* activation of autophagy	(Yang et al., 2018)
JMJD3	Deficiency of JMJD3 and Nox4 prohibits autophagic activation	Attenuates neointima and vascular remodelling following carotid injury	(Luo et al., 2018)
Fra-1	Directly binding and transcriptionally activating p21 and p16 signaling	Promoting vascular aging	(Yang et al., 2019)
GATA4	Directly binding to the the angiogenic factors VEGFA and VEGFC promoter and enhancing transcription.	Regulates Angiogenesis and Persistence of Inflammation	(Jia et al., 2018b)
MCP-1	Hypomethylation of the promoter region in atherosclerosis	Increases the expression of MCP-1, promotes the recruitment of inflammatory cells	(Liu et al., 2012)
eNOS	Hypermethylation of promoter region appears in pathological conditions	Inhibiting the expression of eNOS and NO production	(Chan et al., 2004)
p66Shc	Contains a large number of methylation modification sites	Modifying the methylation level to regulate the gene expression in order to control mitochondrial produce hydrogen peroxide	(Ventura et al., 2002; Cencioni et al., 2013)
miR-217	Combined with the (3’-UTR) of SIRT1 to inhibit the expression of SIRT1	Causing senescence and dysfunction of ECs	(Menghini et al., 2009)
miR-143/miR-145	miR-143 and miR-145 are activated in differentiated smooth muscle cells	Inhibits the proliferation of smooth muscle cells	(Cordes et al., 2009)
miR-210	Reduce the overproduction of ROS	Regulates oxidation stress	(Ma et al., 2018)
miR-135a/miR-714/miR-762/miR-712	Inhibit the outflow of calcium ions by disrupting Ca2+ efflux proteins NCX1, PMCA1, and NCKX4	Promote VSMC calcification	(Gui et al., 2012)
Long non-coding RNA H19	Decreased expressed along with aging in the adult endothelium	Inhibits STAT3 signaling pathway to regulate endothelial cell senescence	(Hofmann et al., 2019)
Long noncoding RNA MEG3	Impairing miR-128-dependent girdin down regulation	Prevents vascular endothelial cell senescence	(Lan et al., 2019)

SIRT, type III histone deacetylases (Sirtuin); VSMC, vascular smooth muscle cell; H4K16, histone4 Lysine16; KLF2, endothelial cells Kruppel-like factor 2. SOD2, superoxide dismutase 2; HDAC, histone deacetylase; FoxO3a, Forkhead boxO3;MCP-1, monocyte chemoattractant protein-1; P66Shc, member of Shc (src homology and collagen homology) family; 3’-UTR, 3’-untranslated region; eNOS, endothelial nitric oxide synthase; NO, nitric oxide; ROS, reactive oxygen species; JMJD3, histone demethylase; GATA4, a member of GATA zinc-finger transcription factor family; Fra-1, Fos-related antigen1; NF-κB, nuclear factor kappa-B; miR, micro RNA; NCX1, Na^+^-Ca^2+^ exchanger isoform 1; NCKX4, Na^+^/K^+/^Ca^2+^-exchange protein 4; PMCA1, plasma membrane calcium ATPase 1; STAT3, signal transducers and activators of transcription.

### Are Epigenetic Mechanisms Involved in the Regulation of Vascular Cell Senescence?

NAD-dependent deacetylase sirtuin-1 (SIRT1), is the most thoroughly studied member of the Nuclear-localized type III histone deacetylases (Situin) family. This protein is involved at multiple levels during the stages of vascular aging, and plays an important role vascular cell senescence as demonstrated by the following observations: (1) the expression of SIRT1 in VSMCs of mice is decreased with advancing age; (2) smooth muscle-specific knockout of SIRT1 in animals promotes Angiotensin II (Ang II) induced vascular senescence (Chen et al., 2016); (3) SIRT1 is important in the deacetylation of histone H4K16, and that this process inhibits senescence of ECs and is protective against vascular aging (Wan et al., 2014); (4) SIRT1 can increase ECs Kruppel-like factor 2 (KLF2) expression, which causes vascular ECs to enter a “vaso-protective” state (Gracia-Sancho et al., 2010); (5) energy restriction promotes increased SIRT1 expression in VSMCs and tissues and fights abdominal aortic aneurysm (Liu et al., 2016); and finally, (6) nuclear-localized SIRT6 protects telomeres in vascular ECs, tempering reductions in replication capacity and premature cell senescence following DNA damage (Cardus et al., 2013).

ncRNAs, especially miRNAs, are involved in vascular cell senescence. miRNAs are short single-stranded ribonucleic acids that can negatively regulate gene expression by base-pairing to target mRNA and causing mRNA cleavage or translation repression (Carthew and Sontheimer, 2009). For example, the expression of miR-217 in ECs is seen to increase with age and can inhibit the expression of SIRT1. Subsequent reductions in the expression of SIRT1 increases the levels of senescent and dysfunction ECs. Interestingly, miR-217 inhibition reportedly slows the rates of cellular senescence in ECs (Menghini et al., 2009). Similarly, miR-145 and miR-143 can regulate the phenotypic transition of smooth muscle cells during vascular aging and during the differentiation of smooth muscle. The miR-143 and miR-145 work together targeted transcription factors network, such as Klf4 (Kruppel-like factor 4) and myocardin and Elk-1 (member of ETS oncogene family) and, on the one hand, form a positive feedback mechanism to promote the differentiation of smooth muscle cells, and on the other hand, repress the proliferation of smooth muscle cells (Cordes et al., 2009). The lncRNA H19 is expressed in the adult endothelium and is reduced with advanced age. H19 inhibits the STAT3 signaling pathway, a key pathway regulating endothelial cell senescence (Hofmann et al., 2019). Other lcRNAs such as MEG3 prevent miR-128–dependent Girdin down regulation and inhibits vascular endothelial cell senescence (Lan et al., 2019). More recently, our research group has shown that Fos-related antigen 1 (Fra-1) plays an important role in Ang II–induced vascular senescence. Fra-1 expression is dramatically increased in Ang II–induced rat aortic endothelial cell (RAEC) senescence (Yang et al., 2019).

### Epigenetic Regulation of Inflammation in Vascular Aging

Typically, an individuals inflammatory status increases with age (Fulop et al., 2018). Studies on the methylation patterns in the human genome have shown that hypermethylation of DNA appears to correlated with chronic inflammation associated with many aging-related diseases (Cutolo et al., 2014). For example, in pathophysiological conditions such as atherosclerosis, hypomethylation of the promoter region of monocyte chemoattractant protein-1 (MCP-1) leads to increased expression of MCP-1 and promotes the recruitment of inflammatory cells accelerating the disease process (Liu et al., 2012).

In other research, the activation of SIRT1 in smooth muscle cells reduces the stiffness of blood vessels; a process associate with crosstalk with the NF-κB inflammatory signaling pathway and the inhibition of inflammatory signaling (Fry et al., 2016). Loss of functional SIRT1 resulted in hyperacetylated of NF-*_K_*B and drives increased transcription of pro-inflammatory genes. Similarly, cytoplasmic SIRT2 induces the deacetylate of p65 Lys310 and regulates NF-κB–dependent gene expression *viz*. reduces transcription of pro-inflammation genes (Rothgiesser et al., 2019). These finding showing that acetylation status of NF-κB is an important driver of rates of inflammation in cells and tissues. Additional support comes from the knowledge that the inhibition of histone deacetylases (HDAC) increases tumor necrosis factor α (TNF-α) levels, the activation of the NF-κB signaling pathway, and resultant increase in IL-8 expression (Ashburner et al., 2001). Other members of the HDAC family such as HDAC3 have been shown to inhibit the activation of macrophages. In macrophages lacking HDAC3, there is an increased response to stimulation by interleukin 4 (IL-4) that likely drives increased rates of inflammation in blood vessel leading to aging (Mullican et al., 2011). In addition, our research on vascular aging points to a possible role of HDAC4 in mitigating inflammatory responses in the vascular system. HDAC4 appears to play an essential role in vascular inflammation by regulates Ang II–induced autophagy *via* the activation of FoxO3a deacetylation (Yang et al., 2018), work recently communicated as an editorial focusing on cardiovascular epigenetics (Tarun and Antoniades, 2018). Additional research from our group has identified histone demethylase Jumonji domain-containing protein 3 (JMJD3) as a key epigenetic regulator of the inflammatory response in cells (Liu et al., 2018). JMJD3 playing a pivitol role in rheumatoid synovial hyperplasia in rheumatoid arthritis (RA) (Jia et al., 2018a; Wu et al., 2019). Moreover, evidence also points to potential roles for JMJD3 in vascular remodeling (Luo et al., 2018) and in the regulation of the transcription factor GATA4. GATA4 functioning in inflammation persistence and angiogenesis in rheumatoid arthritis (RA) (Jia et al., 2018).

Other mechanisms linking epigenetic processes and inflammatory status include the ncRNA molecule, miR-155. miR-155 is a positive regulator of vascular inflammation, and is abundant in activated macrophages and monocytes, and potentially leaves blood vessels in a chronic inflammatory state. Increased levels of miR-155 induce the expression of MCP-1, and could encourage the recruitment of monocytes to vascular tissues thus exacerbating the inflammatory response (Wu et al., 2014). In contrast, miR-194 has opposing effects in that this miRNA can inhibit inflammation. While the mechanisms for this still required additional research, it is known that miR-194 overexpression can inhibit tumor necrosis factor receptor-associated factor 6 (TRAF6), and this reduces the production of monocyte inflammatory factors (Tian et al., 2015).

### Regulation of Oxidation Stress in Vascular Aging

Links between vascular aging, DNA methylation patterns and oxidative stress are also seen in blood vessels. Regulation in the expression of endothelial nitric oxide synthase (eNOS), which encodes an endogenous nitric oxide synthase and is a source of the vasoactive molecule nitric oxide (NO), is altered by methylation status. Lower methylation patterns in the promoter region of eNOS allow for gene transcription and is therefore important in the production of NO and associated physiological processes involving this gaseous signaling molecule. In pathological situations hypermethylation in the eNOS promoter region inhibits eNOS expression and causes diminished levels of NO (Chan et al., 2004). Other proteins such as p66Shc, a protein associated with endothelial dysfunction, are also regulated *via* methylation status. The p66Shc protein is important in signaling systems linked to the production of hydrogen peroxide (H_2_O_2_), and the gene encoding for this protein is known to contains a large number of methylation sites. Modification of the methylation pattern is thus seen as a means to control the expression of this protein (Ventura et al., 2002; Cencioni et al., 2013).

Potential roles of SIRT1 in mitigating oxidative stress in smooth muscle cells has recently been proposed and suggested to be important in reducing blood vessels stiffness (Fry et al., 2016). Similarly, expression of SIRT3 a protein known to decline in tissues by as much as 40% by the age of 65 also appears to be important in mitigating oxidative stress. In cells lacking SIRT3, the activity of the mitochondrial antioxidant enzyme superoxide dismutase 2 (SOD2) is impaired due to hyperacetylation. Consequently, elevated mitochondrial O_2_^•^ and diminished endothelial NO are observed, leading to an imbalance of redox homeostasis in blood vessels (Dikalova et al., 2017). Building on this area of research are roles for miRNA and associated impacts on redox systems linked to vascular ageing. To date, miR-210 has recently been demonstrated to reduce the overproduction of mitochondrial reactive oxygen species (ROS) (Ma et al., 2018).

### Epigenetics Systems and Calcification in Vascular Aging

SIRT1 can inhibit vascular remodeling, stiffness, and functions in protection against atherosclerosis and vascular calcification in mice and is indicative that SIRT1 has protective roles in vascular injury diseases (Winnik et al., 2015). Evidence to support protective roles come from several important pieces of research. Firstly, that *in vitro* culture of VSMCs using media containing high levels of phosphate (Pi) stimulates cell senescence and calcification. These changes related to the down-regulation of SIRT1 expression and the activation of p21(WAF1/Cip1). Activation of p21(WAF1/Cip1) drives replicative senescent in VSMC cells, a process that can be reversed in cells in which p21(WAF1/Cip1) has been knockdown using molecular approaches. Moreover, loss of functional p21(WAF1/Cip1) abolishes Pi induced senescence and calcification in VSCMs. Thirdly, knockdown of SIRT1 in cells promotes a transformation to a calcification phenotype and promotes Pi-induced VSMC senescence calcification. Interestingly, treatment of cells with the SIRT1 induce resveratrol activates the protein and inhibits VSMC calcification (Takemura et al., 2011). In allied areas of research, the expression of the miRNA molecule miR-34a is elevated in the aorta of aged mice and is associated with rates of calcification. miR-34a is downregulating by SIRT1, this serving to temper miR-34a induced VSMCs calcification (Badi et al., 2018). Futhermore, the lncRNA-ES3/miR-34c-5p/BMF axis has recently been shown to regulate high-glucose-induced VSMCs calcification/senescence (Lin et al., 2019), and miR-135a, miR-714, miR-762, and miR-712 are involved in VSMC calcification by disrupting Ca^2+^ efflux proteins like NCX1, PMCA1, and NCKX4 (Gui et al., 2012).

## Epigenetics Regulation and Treatment of Vascular Aging and CVD

As discussed in the previous sections, vascular aging contributes to cardiovascular disease(s) including atherosclerosis, hypertension, coronary heart diseases, and stroke. Vascular aging encompasses many biochemical and physiological changes associated with vascular remodeling, vascular homeostasic imbalance, vascular cell senescence, a pro-inflammation state and increased rates of oxidative stress, and tissue calcification. The interplay between each of these conditions is complex and makes the development of robust treatment strategies targetting vascular aging challenging. Indeed, the targeting of a single vascular cell type or population or a specific signaling system is difficult. However, the recognized association between epigenetic regulation of multiple gene targets coding for proteins regulating biochemical or physiological processes linked to vascular aging may be achievable. Epigenetic targets could serve as appropriate therapeutic targets suitable for the management of vascular aging and related diseases in humans. These treatments, if explored and developed further, may be more effective, span several risk factors linked to the development of vascular aging, and would have the added benefit that they are reversible processes. Such systems could be exploited in the future development of novel therapeutics. For example, our research has demonstrated that JMJD3 could be a useful therapeutic target. JMJD3 is a crucial epigenetic regulator involved in the inflammatory response to LPS in macrophages. JMJD3 expression is controlled by the transcription factor Sp-1 and is responsible for changes in the expression of cystathionine gamma-lyase (CSE). This system negatively regulates the inflammatory response in cells and tissues and reduce the progression of rheumatoid arthritis (RA). Moreover, deficiency of JMJD3 reduces neointima formation after vascular injury by inhibits the Nox4-autophagy signaling pathway. These observations suggesting that JMJD3 may represent a novel target for the development of new anti-inflammatory therapeutics for treating RA, the prevention and treatment of intima hyperplasia-related vascular diseases, and other pro-inflammatory conditions (Liu et al., 2018; Jia et al., 2018a; Luo et al., 2018; Wu et al., 2019). Likewise, the transcription factor GATA4, a key regulator of angiogenesis and persistence of inflammation in RA may also hold promise as a therapeutic target (Jia et al., 2018). We also show that Fos-related antigen 1 (Fra-1) plays a novel and key role in promoting vascular aging by directly binding and activating the target proteins p21(WAF1/Cip1) and p16(INK4A) protein signaling systems. Intervention of Fra-1 is a potential strategy for the prevention of aging-related cardiovascular disorders (Yang et al., 2019).

The development of epigenetically targeted therapeutics has received considerable attention over the last decade and has lead to the identification of several important epigenetic modified protein inhibitors including the FDA-approved molecule azacytidine and various inhibitors of DNMT1, HDAC, and histone acetyltransferases (HAT) (Voelter-Mahlknecht, 2016). These therapeutics could be adopted for use in the treatment of specific CVD conditions. To date, epidrugs-based therapeutics for the treatment of CVD mainly include compounds widely used in the clinical that function through epigenetics-related mechanisms, numerous natural compounds, and various newly synthesized molecules. Our group has reported on the anti-inflammation effects of the HDAC4 inhibitor Tasquinimod and its use in the treatment of vascular inflammation-related diseases (Yang et al., 2018). Other molecules of interest include common statins used to lower serum cholesterol to prevent major cardiovascular problems. Some statins may function as HDAC inhibitor (Voelter-Mahlknecht, 2016). Likewise, trichostatin A, an inhibitor of HDAC, prevents ventricular remodeling by inhibiting TNF-α transcription and by promoting cardiomyocyte survival by enhancing Akt phosphorylation (Zhang et al., 2012). In addition, in experimental models of myocardial infarction and atherosclerosis, the HDAC inhibitor sodium butyrate inhibits NF-κB signal transduction and the production of inflammatory molecules including TNF-α, interleukin-6, vascular cell adhesion molecule-1, and intercellular adhesion molecule-1, pointing to potential pharmacological effects (Hu et al., 2014). The natural product curcumin functions as an HAT inhibitor in rodent models of heart failure preserving systolic function and preventing ventricular hypertrophy (Pan et al., 2013). Similarly, molecules like folic acid and B vitamins are DNMT inhibitors and deficiencies in folic acid causes global DNA hypomethylation that is associated with increased risk of CVD including coronary heart disease, atherosclerosis, and anemia (Kim et al., 2007; McNulty et al., 2008). The common analgesic, acetylsalicylic acid appears to reduces ATP-binding cassette transporter A1 gene methylation rates in the pathophysiology conditions associated with coronary heart disease and thus points to a potentially new therapeutic strategy for this disease (Guay et al., 2014). Likewise, the molecule 5- aza-2-deoxycytidinede (DAC), an inhibitor of DNMT that can reverse rates of DNA methylation, has been shown to re-active genes silenced by hypermethylation *viz*. estrogen receptors α and β in normal ECs and smooth muscle. Importantly, the failure of some estrogen therapies to protect cardiac tissues from damage could be due to epigenetic silencing of the female estrogen receptor. The discovery of natural products that can be used to alter SIRT1 activity in cells has also gained some interest. SIRT1 expression and activity can reduce during senescence-related diseases and re-activation of SIRT1 in tissues may offer new opportunities in the development of future drug candidates. Indeed, one potential drug for disease intervention, is the stilbene resveratrol, an activator of SIRT1. Resveratrol has been repeatedly shown to effectively delay vascular senescence in mice, and to improve cardiometabolic health (da Luz et al., 2012; Pollack and Crandall, 2013; Kim et al., 2018). Finally, rapamycin-induced miR-30a down-regulation is mediated *via* the targeting of beclin1 and can inhibit the senescence of VSMCs (Tan et al., 2019). It is clear that more research is needed to further address the anti-aging effects of many of these molecules and in the discovery of other compounds that could be used to manipulate epigenetic systems in mammalian cells and tissues. In particular, pharmacologist should focus on research to assess the role of other types of epigenetic targeting drugs. Identified molecules could then drive developments in combined approaches to treat CVD using epigenetic-based therapeutics coupled with hormone replacement therapy (Schiano et al., 2015).

## Conclusion and Prospects

Our understanding of the molecular mechanism controlling the epigenetic regulation of gene expression has progressed significantly over the last two decades. However, much has still to be learnt, and our perceived ideas of epigenetic modulation and its manipulation *in vivo* is far more complicated than previously thought. Aging is an irreversible biological process while epigenetic alternations are reversible and may offer novel treatment strategies in patients with age-related CVD. New methods and experimental research techniques are needed to facilitate the manipulation of epigenetic processes in cells and tissues. Indeed, recently a newly emerging epigenetic mechanism involving RNA methylation has been reported (Yue et al., 2015); however, its role in vascular biology is not yet clear and requires further research. Another important problem to be solved in the future is how to manipulate histone modification in specific tissues like the vascular endothelium. This problem is critical because systemic inhibition or activation of HDAC, or other epigenetic enzymes may cause adverse reactions (Heerboth et al., 2014). Building on these advances will be the ability to monitor epigenetic changes in cells, this will be critical in making advances in this field. Interestingly, the progressive development of single-cell sequencing and single-cell epigenetic technologies like scATAC-seq, scDNase-seq, and scChic-seq can be used to study the mode of epigenetic regulation at the single-cell level, and these technologies will offers exciting opportunities in the near future (Nawy, 2014; Ku et al., 2019). In particular, these systems will aid in the development of more elaborate models of epigenetic regulation and will allow for the development of more accurately therapeutics for use in epigenetic research. With regards to vascular aging, a good start here would be research on newer epigenetic pharmaceuticals, developed using drug repurposing approaches; a safe and low-cost way to support future vascular drug discovery (Xu et al., 2019). In addition, since epigenetic mechanisms work in concert to regulate gene networks, there may also be requirements for the development of epigenetic “cocktail” therapies that can be exploited to target a spectrum of age-related genes for treating age-related diseases.

## Author Contributions

All authors contributed to the article and approved the submitted version.

## Funding

This work was supported by the National Natural Science Foundation of China (81973320), Macau Science and Technology Development fund (FDCT) (067/2018/A2,033/2017/AMJ, 0007/2019/AKP, 0052/2020/A), and the Key Laboratory Program of the Education Commission of Shanghai Municipality (no. ZDSYS14005).

## Conflict of Interest

The authors declare that the research was conducted in the absence of any commercial or financial relationships that could be construed as a potential conflict of interest.

## References

[B1] Anderson K. M. Anderson D. M. McAnally J. R. Shelton J. M. Bassel-Duby R. Olson E. N. (2016). Transcription of the non-coding RNA upperhand controls Hand2 expression and heart development. Nature 539 (7629), 433–436. 10.1038/nature20128 27783597 PMC5261552

[B2] Ashburner B. P. Westerheide S. D. Baldwin A. S. Jr (2001). The p65 (RelA) subunit of NF-kappaB interacts with the histone deacetylase (HDAC) corepressors HDAC1 and HDAC2 to negatively regulate gene expression. Mol. Cell Biol. 21 (20), 7065–7077. 10.1128/MCB.21.20.7065-7077.2001 11564889 PMC99882

[B3] Badi I. Mancinelli L. Polizzotto A. Ferri D. Zeni F. Burba I. (2018). miR-34a Promotes Vascular Smooth Muscle Cell Calcification by Downregulating SIRT1 (Sirtuin 1) and Axl (AXL Receptor Tyrosine Kinase). Arterioscler. Thromb. Vasc. Biol. 38 (9), 2079–2090. 10.1161/ATVBAHA.118.311298 30026277

[B4] Calvanese V. Lara E. Kahn A. Fraga M. F. (2009). The role of epigenetics in aging and age-related diseases. Ageing Res. Rev. 8 (4), 268–276. 10.1016/j.arr.2009.03.004 19716530

[B5] Cardus A. Uryga A. K. Walters G. Erusalimsky J. D. (2013). SIRT6 protects human endothelial cells from DNA damage, telomere dysfunction, and senescence. Cardiovasc. Res. 97 (3), 571–579. 10.1093/cvr/cvs352 23201774 PMC3567786

[B6] Carthew R. W. Sontheimer E. J. (2009). Origins and Mechanisms of miRNAs and siRNAs. Cell 136 (4), 642–655. 10.1016/j.cell.2009.01.035 19239886 PMC2675692

[B7] Cavallaro U. Castelli V. Del Monte U. Soria M. R. (2000). Phenotypic alterations in senescent large-vessel and microvascular endothelial cells. Mol. Cell Biol. Res. Commun. 4 (2), 117–121. 10.1006/mcbr.2000.0263 11170842

[B8] Cencioni C. Spallotta F. Martelli F. Valente S. Mai A. Zeiher A. M. (2013). Oxidative stress and epigenetic regulation in ageing and age-related diseases. Int. J. Mol. Sci. 14 (9), 17643–17663. 10.3390/ijms140917643 23989608 PMC3794746

[B9] Chan Y. Fish J. E. D’Abreo C. Lin S. Robb G. B. Teichert A. M. (2004). The cell-specific expression of endothelial nitric-oxide synthase: a role for DNA methylation. J. Biol. Chem. 279 (33), 35087–35100. 10.1074/jbc.M405063200 15180995

[B10] Chen H. Z. Wang F. Gao P. Pei J. F. Liu Y. Xu T. T. (2016). Age-Associated Sirtuin 1 Reduction in Vascular Smooth Muscle Links Vascular Senescence and Inflammation to Abdominal Aortic Aneurysm. Circ. Res. 119 (10), 1076–1088. 10.1161/CIRCRESAHA.116.308895 27650558 PMC6546422

[B11] Cordes K. R. Sheehy N. T. White M. P. Berry E. C. Morton S. U. Muth A. N. (2009). miR-145 and miR-143 regulate smooth muscle cell fate and plasticity. Nature 460 (7256), 705–710. 10.1038/nature08195 19578358 PMC2769203

[B12] Cunha P. G. Boutouyrie P. Nilsson P. M. Laurent S. (2017). Early Vascular Ageing (EVA): Definitions and Clinical Applicability. Curr. Hypertens. Rev. 13 (1), 8–15. 10.2174/1573402113666170413094319 28412914

[B13] Cutolo M. Paolino S. Pizzorni C. (2014). Possible contribution of chronic inflammation in the induction of cancer in rheumatic diseases. Clin. Exp. Rheumatol. 32 (6), 839–847. 10.3177/jnsv.47.385 25496746

[B14] da Luz P. L. Tanaka L. Brum P. C. Dourado P. M. Favarato D. Krieger J. E. (2012). Red wine and equivalent oral pharmacological doses of resveratrol delay vascular aging but do not extend life span in rats. Atherosclerosis 224 (1), 136–142. 10.1016/j.atherosclerosis.2012.06.007 22818625

[B15] Dikalova A. E. Itani H. A. Nazarewicz R. R. McMaster W. G. Flynn C. R. Uzhachenko R. (2017). Sirt3 Impairment and SOD2 Hyperacetylation in Vascular Oxidative Stress and Hypertension. Circ. Res. 121 (5), 564–574. 10.1161/CIRCRESAHA.117.310933 28684630 PMC5562527

[B16] Ding Y. N. Tang X. Chen H. Z. Liu D. P. (2018). Epigenetic Regulation of Vascular Aging and Age-Related Vascular Diseases. Adv. Exp. Med. Biol. 1086, 55–75. 10.1007/978-981-13-1117-8_4 30232752

[B17] Dzau V. J. Antman E. M. Black H. R. Hayes D. L. Manson J. E. Plutzky J. (2006a). The cardiovascular disease continuum validated: clinical evidence of improved patient outcomes: part I: Pathophysiology and clinical trial evidence (risk factors through stable coronary artery disease). Circulation 114 (25), 2850–2870. 10.1161/CIRCULATIONAHA.106.655688 17179034

[B18] Dzau V. J. Antman E. M. Black H. R. Hayes D. L. Manson J. E. Plutzky J. (2006b). The cardiovascular disease continuum validated: clinical evidence of improved patient outcomes: part II: Clinical trial evidence (acute coronary syndromes through renal disease) and future directions. Circulation 114 (25), 2871–2891. 10.1161/CIRCULATIONAHA.106.655761 17179035

[B19] Eto H. Miyata M. Shirasawa T. Akasaki Y. Hamada N. Nagaki A. (2008). The long-term effect of angiotensin II type 1a receptor deficiency on hypercholesterolemia-induced atherosclerosis. Hypertens. Res. 31 (8), 1631–1642. 10.1291/hypres.31.1631 18971539

[B20] Feinberg A. P. (2018). The Key Role of Epigenetics in Human Disease Prevention and Mitigation. N. Engl. J. Med. 378 (14), 1323–1334. 10.1056/NEJMra1402513 29617578 PMC11567374

[B21] Fraga M. F. Ballestar E. Paz M. F. Ropero S. Setien F. Ballestar M. L. (2005). Epigenetic differences arise during the lifetime of monozygotic twins. Proc. Natl. Acad. Sci. U. S. A. 102 (30), 10604–10609. 10.1073/pnas.0500398102 16009939 PMC1174919

[B22] Fry J. L. Al Sayah L. Weisbrod R. M. VanRoy I. Weng X. Cohen R. A. (2016). Vascular Smooth Muscle Sirtuin-1 Protects Against Diet-Induced Aortic Stiffness. Hypertension 68 (3), 775–784. 10.1161/HYPERTENSIONAHA.116.07622 27432859 PMC4982825

[B23] Fu Z. Wang M. Gucek M. Zhang J. Wu J. Jiang L. (2009). Milk fat globule protein epidermal growth factor-8: a pivotal relay element within the angiotensin II and monocyte chemoattractant protein-1 signaling cascade mediating vascular smooth muscle cells invasion. Circ. Res. 104 (12), 1337–1346. 10.1161/CIRCRESAHA.108.187088 19443842 PMC2764993

[B24] Fulop T. Larbi A. Dupuis G. LePage A. Frost E. H. Cohen A. A. (2018). Immunosenescence and Inflamm-Aging As Two Sides of the Same Coin: Friends or Foes? Front. Immunol. 8:1960. 10.3389/fimmu.2017.01960 29375577 PMC5767595

[B25] Gracia-Sancho J. Villarreal G. Jr Zhang Y. García-Cardeña G. (2010). Activation of SIRT1 by resveratrol induces KLF2 expression conferring an endothelial vasoprotective phenotype. Cardiovasc. Res. 85 (3), 514–519. 10.1093/cvr/cvp337 19815564 PMC2802207

[B26] Guay S. P. Légaré C. Houde A. A. Mathieu P. Bossé Y. Bouchard L. (2014). Acetylsalicylic acid, aging and coronary artery disease are associated with ABCA1 DNA methylation in men. Clin. Epigenet. 6 (1):14. 10.1186/1868-7083-6-14 PMC4120725 25093045

[B27] Gui T. Zhou G. Sun Y. Shimokado A. Itoh S. Oikawa K. (2012). MicroRNAs that target Ca(2+) transporters are involved in vascular smooth muscle cell calcification. Lab. Invest. 92 (9), 1250–1259. 10.1038/labinvest.2012.85 22688076

[B28] Gurha P. Marian A. J. (2013). Noncoding RNAs in cardiovascular biology and disease. Circ. Res. 113 (12), e115–e120. 10.1161/CIRCRESAHA.113.302988 24311620

[B29] Hai Z. Zuo W. (2016). Aberrant DNA methylation in the pathogenesis of atherosclerosis. Clin. Chim. Acta 456, 69–74. 10.1016/j.cca.2016.02.026 26944567

[B30] Handy D. E. Castro R. Loscalzo J. (2011). Epigenetic modifications: basic mechanisms and role in cardiovascular disease. Circulation 123 (19), 2145–2156. 10.1161/CIRCULATIONAHA.110.956839 21576679 PMC3107542

[B31] Heerboth S. Lapinska K. Snyder N. Leary M. Rollinson S. Sarkar S. (2014). Use of epigenetic drugs in disease: an overview. Genet. Epigenet. 6, 9–19. 10.4137/GEG.S12270 25512710 PMC4251063

[B32] Hofmann P. Sommer J. Theodorou K. Kirchhof L. Fischer A. Li Y. (2019). Long non-coding RNA H19 regulates endothelial cell aging via inhibition of STAT3 signalling. Cardiovasc. Res. 115 (1), 230–242. 10.1093/cvr/cvy206 30107531 PMC6302267

[B33] Hu X. Zhang K. Xu C. Chen Z. Jiang H. (2014). Anti-inflammatory effect of sodium butyrate preconditioning during myocardial ischemia/reperfusion. Exp. Ther. Med. 8 (1), 229–232. 10.3892/etm.2014.1726 24944626 PMC4061237

[B34] Jeltsch A. (2008). Reading and writing DNA methylation. Nat. Struct. Mol. Biol. 15 (10), 1003–1004. 10.1038/nsmb1008-1003 18836494

[B35] Jia W. Wu W. Yang D. Xiao C. Su Z. Huang Z. (2018a). Histone demethylase JMJD3 regulates fibroblast-like synoviocyte-mediated proliferation and joint destruction in rheumatoid arthritis. FASEB J. 32 (7), 4031–4042. 10.1096/fj.201701483R 29481307

[B36] Jia W. Wu W. Yang D. Xiao C. Huang M. Long F. (2018b). GATA4 regulates angiogenesis and persistence of inflammation in rheumatoid arthritis. Cell Death Dis. 9 (5), 503. 10.1038/s41419-018-0570-5 29717129 PMC5931571

[B37] Jiang L. Zhang J. Monticone R. E. Telljohann R. Wu J. Wang M. (2012). Calpain-1 regulation of matrix metalloproteinase 2 activity in vascular smooth muscle cells facilitates age-associated aortic wall calcification and fibrosis. Hypertension 60 (5), 1192–1199. 10.1161/HYPERTENSIONAHA.112.196840 23006733 PMC3487400

[B38] Kadlec A. O. Chabowski D. S. Ait-Aissa K. Gutterman D. D. (2016). Role of PGC-1α in Vascular Regulation: Implications for Atherosclerosis. Arterioscler. Thromb. Vasc. Biol. 36 (8), 1467–1474. 10.1161/ATVBAHA.116.307123 27312223 PMC4965312

[B39] Kim J. Kim J. Y. Song K. S. Lee Y. H. Seo J. S. Jelinek J. (2007). Epigenetic changes in estrogen receptor beta gene in atherosclerotic cardiovascular tissues and in-vitro vascular senescence. Biochim. Biophys. Acta 1772 (1), 72–80. 10.1016/j.bbadis.2006.10.004 17110088

[B40] Kim E. N. Kim M. Y. Lim J. H. Kim Y. Shin S. J. Park C. (2018). The protective effect of resveratrol on vascular aging by modulation of the renin-angiotensin system. Atherosclerosis 270, 123–131. 10.1016/j.atherosclerosis.2018.01.043 29407880

[B41] Kohli R. M. Zhang Y. (2013). TET enzymes, TDG and the dynamics of DNA demethylation. Nature 502 (7472), 472–479. 10.1038/nature12750 24153300 PMC4046508

[B42] Krebs J. Agellon L. B. Michalak M. (2015). Ca(2+) homeostasis and endoplasmic reticulum (ER) stress: An integrated view of calcium signaling. Biochem. Biophys. Res. Commun. 460 (1), 114–121. 10.1016/j.bbrc.2015.02.004 25998740

[B43] Ku W. L. Nakamura K. Gao W. Cui K. Hu G. Tang Q. (2019). Single-cell chromatin immunocleavage sequencing (scChIC-seq) to profile histone modification. Nat. Methods 16 (4), 323–325. 10.1038/s41592-019-0361-7 30923384 PMC7187538

[B44] Lakatta E. G. Levy D. (2003a). Arterial and cardiac aging: major shareholders in cardiovascular disease enterprises: Part I: aging arteries: a “set up” for vascular disease. Circulation 107 (1), 139–146. 10.1161/01.cir.0000048892.83521.58 12515756

[B45] Lakatta E. G. Levy D. (2003b). Arterial and cardiac aging: major shareholders in cardiovascular disease enterprises: Part II: the aging heart in health: links to heart disease. Circulation 107 (2), 346–354. 10.1161/01.cir.0000048893.62841.f7 12538439

[B46] Lakatta E. G. Wang M. Najjar S. S. (2009). Arterial aging and subclinical arterial disease are fundamentally intertwined at macroscopic and molecular levels. Med. Clin. North Am. 93 (3), 583–Contents. 10.1016/j.mcna.2009.02.008 19427493 PMC2943242

[B47] Lakatta E. G. (2003). Arterial and cardiac aging: major shareholders in cardiovascular disease enterprises: Part III: cellular and molecular clues to heart and arterial aging. Circulation 107 (3), 490–497. 10.1161/01.cir.0000048894.99865.02 12551876

[B48] Lan Y. Li Y. J. Li D. J. Li P. Wang J. Y. Diao Y. P. (2019). Long noncoding RNA MEG3 prevents vascular endothelial cell senescence by impairing miR-128-dependent Girdin downregulation. Am. J. Physiol. Cell Physiol. 316 (6), C830–C843. 10.1152/ajpcell.00262.2018 30576236

[B49] Leopold J. A. (2013). Vascular calcification: an age-old problem of old age. Circulation 127 (24), 2380–2382. 10.1161/CIRCULATIONAHA.113.003341 23690467 PMC3761061

[B50] Lin X. Zhan J. K. Zhong J. Y. Wang Y. J. Wang Y. Li S. (2019). lncRNA-ES3/miR-34c-5p/BMF axis is involved in regulating high-glucose-induced calcification/senescence of VSMCs. Aging (Albany N. Y.) 11 (2), 523–535. 10.18632/aging.101758 PMC6366973 30654331

[B51] Liu X. L. Zhang P. F. Ding S. F. Wang Y. Zhang M. Zhao Y. X. (2012). Local gene silencing of monocyte chemoattractant protein-1 prevents vulnerable plaque disruption in apolipoprotein E-knockout mice. PLoS One 7 (3), e33497. 10.1371/journal.pone.0033497 22428064 PMC3299803

[B52] Liu Y. Wang T. T. Zhang R. Fu W. Y. Wang X. Wang F. (2016). Calorie restriction protects against experimental abdominal aortic aneurysms in mice. J. Exp. Med. 213 (11), 2473–2488. 10.1084/jem.20151794 27670594 PMC5068228

[B53] Liu S. Wang X. Pan L. Wu W. Yang D. Qin M. (2018). Endogenous hydrogen sulfide regulates histone demethylase JMJD3-mediated inflammatory response in LPS-stimulated macrophages and in a mouse model of LPS-induced septic shock. Biochem. Pharmacol. 149, 153–162. 10.1016/j.bcp.2017.10.010 29074105

[B54] Luo X. Yang D. Wu W. Long F. Xiao C. Qin M. (2018). Critical role of histone demethylase Jumonji domain-containing protein 3 in the regulation of neointima formation following vascular injury. Cardiovasc. Res. 114 (14), 1894–1906. 10.1093/cvr/cvy176 29982434

[B55] Ma X. Wang J. Li J. Ma C. Chen S. Lei W. (2018). Loading MiR-210 in Endothelial Progenitor Cells Derived Exosomes Boosts Their Beneficial Effects on Hypoxia/Reoxygeneation-Injured Human Endothelial Cells via Protecting Mitochondrial Function. Cell Physiol. Biochem. 46 (2), 664–675. 10.1159/000488635 29621777

[B56] Maegawa S. Lu Y. Tahara T. Lee J. T. Madzo J. Liang S. (2017). Caloric restriction delays age-related methylation drift. Nat. Commun. 8 (1), 539. 10.1038/s41467-017-00607-3 28912502 PMC5599616

[B57] McGill H. C. Jr McMahan C. A. Gidding S. S. (2008). Preventing heart disease in the 21st century: implications of the Pathobiological Determinants of Atherosclerosis in Youth (PDAY) study. Circulation 117 (9), 1216–1227. 10.1161/CIRCULATIONAHA.107.717033 18316498

[B58] McNulty H. Pentieva K. Hoey L. Ward M. (2008). Homocysteine, B-vitamins and CVD. Proc. Nutr. Soc 67 (2), 232–237. 10.1017/S0029665108007076 18412997

[B59] Menghini R. Casagrande V. Cardellini M. Martelli E. Terrinoni A. Amati F. (2009). MicroRNA 217 modulates endothelial cell senescence via silent information regulator 1. Circulation 120 (15), 1524–1532. 10.1161/CIRCULATIONAHA.109.864629 19786632

[B60] Mikhed Y. Daiber A. Steven S. (2015). Mitochondrial Oxidative Stress, Mitochondrial DNA Damage and Their Role in Age-Related Vascular Dysfunction. Int. J. Mol. Sci. 16 (7), 15918–15953. 10.3390/ijms160715918 26184181 PMC4519931

[B61] Mullican S. E. Gaddis C. A. Alenghat T. Nair M. G. Giacomin P. R. Everett L. J. (2011). Histone deacetylase 3 is an epigenomic brake in macrophage alternative activation. Genes Dev. 25 (23), 2480–2488. 10.1101/gad.175950.111 22156208 PMC3243058

[B62] Nawy T. (2014). Single-cell sequencing. Nat. Methods 11 (1), 18. 10.1038/nmeth.2771 24524131

[B63] Novella S. Heras M. Hermenegildo C. Dantas A. P. (2012). Effects of estrogen on vascular inflammation: a matter of timing. Arterioscler. Thromb. Vasc. Biol. 32 (8), 2035–2042. 10.1161/ATVBAHA.112.250308 22679310

[B64] Okano M. Bell D. W. Haber D. A. Li E. (1999). DNA methyltransferases Dnmt3a and Dnmt3b are essential for de novo methylation and mammalian development. Cell 99 (3), 247–257. 10.1016/s0092-8674(00)81656-6 10555141

[B65] O’Rourke M. F. Safar M. E. Dzau V. (2010). The Cardiovascular Continuum extended: aging effects on the aorta and microvasculature. Vasc. Med. 15 (6), 461–468. 10.1177/1358863X10382946 21056945

[B66] Pan M. H. Lai C. S. Wu J. C. Ho C. T. (2013). Epigenetic and disease targets by polyphenols. Curr. Pharm. Des. 19 (34), 6156–6185. 10.2174/1381612811319340010 23448446

[B67] Pollack R. M. Crandall J. P. (2013). Resveratrol: therapeutic potential for improving cardiometabolic health. Am. J. Hypertens. 26 (11), 1260–1268. 10.1093/ajh/hpt165 24025725

[B68] Rothgiesser K. M. Erener S. Waibel S. Lüscher B. Hottiger M. O. (2019). Correction: SIRT2 regulates NF-κB-dependent gene expression through deacetylation of p65 Lys310 (doi:10.1242/jcs.073783). J. Cell Sci. 132 (8), jcs232801. 10.1242/jcs.232801 31018986

[B69] Schiano C. Vietri M. T. Grimaldi V. Picascia A. De Pascale M. R. Napoli C. (2015). Epigenetic-related therapeutic challenges in cardiovascular disease. Trends Pharmacol. Sci. 36 (4), 226–235. 10.1016/j.tips.2015.02.005 25758254

[B70] Sen P. Shah P. P. Nativio R. Berger S. L. (2016). Epigenetic Mechanisms of Longevity and Aging. Cell 166 (4), 822–839. 10.1016/j.cell.2016.07.050 27518561 PMC5821249

[B71] Shahbazian M. D. Grunstein M. (2007). Functions of site-specific histone acetylation and deacetylation. Annu. Rev. Biochem. 76, 75–100. 10.1146/annurev.biochem.76.052705.162114 17362198

[B72] Takemura A. Iijima K. Ota H. Son B. K. Ito Y. Ogawa S. (2011). Sirtuin 1 retards hyperphosphatemia-induced calcification of vascular smooth muscle cells. Arterioscler. Thromb. Vasc. Biol. 31 (9), 2054–2062. 10.1161/ATVBAHA.110.216739 21719763

[B73] Tan Q. Christiansen L. Thomassen M. Kruse T. A. Christensen K. (2013). Twins for epigenetic studies of human aging and development. Ageing Res. Rev. 12 (1), 182–187. 10.1016/j.arr.2012.06.004 22750314 PMC3509237

[B74] Tan P. Wang H. Zhan J. Ma X. Cui X. Wang Y. (2019). Rapamycinߛinduced miRߛ30a downregulation inhibits senescence of VSMCs by targeting Beclin1. Int. J. Mol. Med. 43 (3), 1311–1320. 10.3892/ijmm.2019.4074 30747228 PMC6365076

[B75] Tarun A. Antoniades C. (2018). The era of cardiovascular epigenetics: histone deacetylases and vascular inflammation. Cardiovasc. Res. 114 (7), 928–930. 10.1093/cvr/cvy099 29718135

[B76] Tian H. Liu C. Zou X. Wu W. Zhang C. Yuan D. (2015). MiRNA-194 Regulates Palmitic Acid-Induced Toll-Like Receptor 4 Inflammatory Responses in THP-1 Cells. Nutrients 7 (5), 3483–3496. 10.3390/nu7053483 25984739 PMC4446763

[B77] Tsaprouni L. G. Yang T. P. Bell J. Dick K. J. Kanoni S. Nisbet J. (2014). Cigarette smoking reduces DNA methylation levels at multiple genomic loci but the effect is partially reversible upon cessation. Epigenetics 9 (10), 1382–1396. 10.4161/15592294.2014.969637 25424692 PMC4623553

[B78] Van Camp G. (2014). Cardiovascular disease prevention. Acta Clin. Belg. 69 (6), 407–411. 10.1179/2295333714Y.0000000069 25176558

[B79] Ventura A. Luzi L. Pacini S. Baldari C. T. Pelicci P. G. (2002). The p66Shc longevity gene is silenced through epigenetic modifications of an alternative promoter. J. Biol. Chem. 277 (25), 22370–22376. 10.1074/jbc.M200280200 11948181

[B80] Voelter-Mahlknecht S. (2016). Epigenetic associations in relation to cardiovascular prevention and therapeutics. Clin. Epigenet. 8, 4. 10.1186/s13148-016-0170-0 PMC4714496 26779291

[B81] Wan Y. Z. Gao P. Zhou S. Zhang Z. Q. Hao D. L. Lian L. S. (2014). SIRT1-mediated epigenetic downregulation of plasminogen activator inhibitor-1 prevents vascular endothelial replicative senescence. Aging Cell 13 (5), 890–899. 10.1111/acel.12247 25040736 PMC4331759

[B82] Wang M. Khazan B. Lakatta E. G. (2010a). Central Arterial Aging and Angiotensin II Signaling. Curr. Hypertens. Rev. 6 (4), 266–281. 10.2174/157340210793611668 21423831 PMC3058527

[B83] Wang M. Monticone R. E. Lakatta E. G. (2010b). Arterial aging: a journey into subclinical arterial disease. Curr. Opin. Nephrol. Hypertens. 19 (2), 201–207. 10.1097/MNH.0b013e3283361c0b 20040868 PMC2943205

[B84] Winnik S. Auwerx J. Sinclair D. A. Matter C. M. (2015). Protective effects of sirtuins in cardiovascular diseases: from bench to bedside. Eur. Heart J. 36 (48), 3404–3412. 10.1093/eurheartj/ehv290 26112889 PMC4685177

[B85] Wu X. Y. Fan W. D. Fang R. Wu G. F. (2014). Regulation of microRNA-155 in endothelial inflammation by targeting nuclear factor (NF)-κB P65. J. Cell Biochem. 115 (11), 1928–1936. 10.1002/jcb.24864 24905663

[B86] Wu C. L. Wang Y. Jin B. Chen H. Xie B. S. Mao Z. B. (2015). Senescence-associated Long Non-coding RNA (SALNR) Delays Oncogene-induced Senescence through NF90 Regulation. J. Biol. Chem. 290 (50), 30175–30192. 10.1074/jbc.M115.661785 26491010 PMC4705997

[B87] Wu W. Qin M. Jia W. Huang Z. Li Z. Yang D. (2019). Cystathionine-γ-lyase ameliorates the histone demethylase JMJD3-mediated autoimmune response in rheumatoid arthritis. Cell Mol. Immunol. 16 (8), 694–705. 10.1038/s41423-018-0037-8 29844591 PMC6804949

[B88] Xu S. Kamato D. Little P. J. Nakagawa S. Pelisek J. Jin Z. G. (2019). Targeting epigenetics and non-coding RNAs in atherosclerosis: from mechanisms to therapeutics. Pharmacol. Ther. 196, 15–43. 10.1016/j.pharmthera.2018.11.003 30439455 PMC6450782

[B89] Yang D. Xiao C. Long F. Su Z. Jia W. Qin M. (2018). HDAC4 regulates vascular inflammation via activation of autophagy. Cardiovasc. Res. 114 (7), 1016–1028. 10.1093/cvr/cvy051 29529137

[B90] Yang D. Xiao C. Long F. Wu W. Huang M. Qu L. (2019). Fra-1 plays a critical role in angiotensin II-induced vascular senescence. FASEB J. 33 (6), 7603–7614. 10.1096/fj.201801671RRRR 30892941

[B91] Yue Y. Liu J. He C. (2015). RNA N6-methyladenosine methylation in post-transcriptional gene expression regulation. Genes Dev. 29 (13), 1343–1355. 10.1101/gad.262766.115 26159994 PMC4511210

[B92] Zhang L. Qin X. Zhao Y. Fast L. Zhuang S. Liu P. (2012). Inhibition of histone deacetylases preserves myocardial performance and prevents cardiac remodeling through stimulation of endogenous angiomyogenesis. J. Pharmacol. Exp. Ther. 341 (1), 285–293. 10.1124/jpet.111.189910 22271820 PMC3310703

